# Lasiodin Inhibits Proliferation of Human Nasopharyngeal Carcinoma Cells by Simultaneous Modulation of the Apaf-1/Caspase, AKT/MAPK and COX-2/NF-κB Signaling Pathways

**DOI:** 10.1371/journal.pone.0097799

**Published:** 2014-05-20

**Authors:** Lianzhu Lin, Wuguo Deng, Yun Tian, Wangbing Chen, Jingshu Wang, Lingyi Fu, Dingbo Shi, Mouming Zhao, Wei Luo

**Affiliations:** 1 College of Light Industry and Food Sciences, South China University of Technology, Guangzhou, China; 2 State Key Laboratory of Oncology in South China, Sun Yat-Sen University Cancer Center, Guangzhou, China; 3 State Key Laboratory of Pulp and Paper Engineering, South China University of Technology, Guangzhou, China; 4 Analysis and Test Center of South China University of Technology, Guangzhou, China; Duke University Medical Center, United States of America

## Abstract

*Rabdosia serra* has been widely used for the treatment of the various human diseases. However, the antiproliferative effects and underlying mechanisms of the compounds in this herb remain largely unknown. In this study, an antiproliferative compound against human nasopharyngeal carcinoma (NPC) cells from *Rabdosia serra* was purified and identified as lasiodin (a diterpenoid). The treatment with lasiodin inhibited cell viability and migration. Lasiodin also mediated the cell morphology change and induced apoptosis in NPC cells. The treatment with lasiodin induced the Apaf-1 expression, triggered the cytochrome-C release, and stimulated the PARP, caspase-3 and caspase-9 cleavages, thereby activating the apoptotic pathways. The treatment with lasiodin also significantly inhibited the phosphorylations of the AKT, ERK1/2, p38 and JNK proteins. The pretreatment with the AKT or MAPK-selective inhibitors considerably blocked the lasiodin-mediated inhibition of cell proliferation. Moreover, the treatment with lasiodin inhibited the COX-2 expression, abrogated NF-κB binding to the COX-2 promoter, and promoted the NF-κB translocation from cell nuclei to cytosol. The pretreatment with a COX-2-selective inhibitor abrogated the lasiodin-induced inhibition of cell proliferation. These results indicated that lasiodin simultaneously activated the Apaf-1/caspase-dependent apoptotic pathways and suppressed the AKT/MAPK and COX-2/NF-κB signaling pathways. This study also suggested that lasiodin could be a promising natural compound for the prevention and treatment of NPC.

## Introduction

Nasopharyngeal carcinoma (NPC) is an unique type of the head and neck cancer, which is a common malignancy in southern China and Southeast Asia [1]. NPC is associated with a high metastasis rate. And it is radiosensitive [2]. However, many patients have the metastases to the regional lymph nodes or distant organs at the time of the diagnosis [1]. Nevertheless, the current therapeutic strategies for promoting the local control of advanced NPC led to the acute toxicity and even increased the incidence of the late complications without the survival benefits [2]. Chemotherapy is one of the most extensively studied treatments in anticancer therapies. The uses of the natural, synthetic and biologic chemicals are considered as the effective cancer chemoprevention in the prevention, suppression, and delay of the carcinogenesis process [3]. Therefore, the combination of chemotherapy and radiotherapy for the NPC treatment may enhance the effects of radiotherapy alone. This strategy may be feasible in patients with locally advanced NPC.

Non-toxic phytochemicals are especially attractive for the chemoprevention due to their ability to simultaneously target several events related to carcinogenesis such as apoptosis, inflammation and angiogenesis [4]. Nowadays, non-toxic phytochemicals such as curcumin [5], epigallocatechin gallate [6], and soy isoflavones [7] have been investigated in the various phases of the clinical trials.

*Rabdosia serra* (Maxim.) Hara has been used for the treatment of arthritis, enteritis, jaundice, hepatitis, lepromatous leprosy, ascariasis and acute cholecystitis [8]. *R. serra* is consumed as herbal tea due to its health benefits, unique flavor and pleasant aroma [9]. The previous phytochemical investigations have led to the isolation and identification of the phenolics, diterpenoids and polysaccharides [10]–[15]. A series of *ent*-kaurane-type diterpenoids possessing strong cytotoxicities against human hepatocellular liver carcinoma cells, breast adenocarcinoma cells, and promyelocytic leukemia cells were reported in our previous study [16]. Despite of the various pharmacological activities of the diterpenoids isolated from *R. serra* [17], the molecular mechanisms in tumor cells have not been clearly explained. On the basis of the previous reports and our continuing research interest on *R. serra* with the cancer chemopreventive potential, the present investigation was undertaken to isolate and identify the compounds with antiproliferative effects against NPC cells through an antiproliferative activity-guided assay.

Apoptosis has been demonstrated to play an important role in human cancer development [18]. Several proteins are directly involved in the apoptotic process, including the Bcl-2 family, mitogen-activated protein kinase (MAPK) family, and phosphoinositide 3-kinase (PI3K)/AKT family [19]. The expression of COX-2 plays a critical role in inflammation and tumorigenesis [20]. The abnormal expression of COX-2 can cause the tumor promotion. In addition, the invasiveness, prognosis and survival in some cancers are highly correlated to the overexpression of COX-2 [21]. COX-2 promotes the cell survival mainly through regulating the Bcl-2 family members [22], [23]. To date, the downregulation of the COX-2 expression with the chemotherapy agents is proposed for the cancer treatment. However, it is uncertain whether the active compounds purified from *R. serra* can simultaneously regulate the Bcl-2, AKT/MAPK and COX-2-dependent signaling pathways to inhibit NPC cell proliferation.

In this study, we purified an antiproliferative compound against NPC cells from *R. serra*. The effects of the active compound on proliferation, migration, and apoptosis in NPC cells were measured. We also investigated its effects on some key proteins involved in the growth-dependent signaling pathways to illustrate the underlying molecular mechanisms.

## Materials and Methods

### Plant material

The aerial parts of *R. serra* were collected from Luofu mountain (GPS coordinates: 23.29522, 114.105266), Huizhou, Guangdong, China on September 14^th^, 2011. And they were authenticated by Professor Huagu Ye of South China Botanical Garden, Chinese Academy of Sciences, where voucher specimens (voucher specimen number 21373) were kept. *R. serra* leaf was separated from the stem, cleanly washed without any damage, dried in the sun, and ground into fine powder by a laboratory mill (FW100, Taisite Instrument Co., Ltd, Tianjin, China). No specific permissions were required for these locations or activities. In addition, the field studies did not involve endangered or protected species.

### Cell culture

The human NPC cell lines CNE1 (human nasopharyngeal high differentiated squamous epithelium carcinoma cell) and CNE2 (human nasopharyngeal low differentiated squamous epithelium carcinomas cell) were purchased from American Type Culture Collection (ATCC). NPC cells were cultured in the DMEM medium (Invitrogen, Carlsbad, CA), supplemented with 10% fetal bovine serum (FBS) (HyClone, Logan, UT), and maintained in an incubator with a humidified atmosphere of 95% air and 5% CO_2_ at 37°C.

### Reagents

LY294002, SB203580, SP600125, U0126, celecoxib and streptavidin-agarose were purchased from Sigma (St. Louis, MO).

### Cell viability assay

Cell viability was determined by the MTT assay. Briefly, NPC cells plated in the 96-well plates (2000 cells/well) were treated with the samples at the indicated doses. NPC cells treated with the vehicle control (DMSO) were used as the reference group. The final concentration of DMSO in the cell culture was 1%. At 24, 48, or 72 hr after the treatment, cell viability was determined.

### Chemical analysis methods

1D NMR spectra were recorded on a Bruker AVANCE spectrometer (Bruker DRX400, Bruker Biospin Co., Karlsruhe, Germany). Mass spectra were recorded on an electrospray ionization (ESI) mass spectrometry system (LCQDECA, Finigan Inc., CA, USA). Column chromatography was performed on silica gel (200–300 mesh; Qingdao Marine Chemical Inc., Qingdao, China), ODS-BP gel (Daiso Co. Ltd., Osaka, Japan), and Sephadex LH-20 gel (Pharmacia Fine Chemicals, Uppsala, Sweden). HPLC analysis was carried out using a Waters 600 HPLC system (Waters, Milford, MA, USA) as previously described [10].

### The antiproliferative activity-guided purification and identification

**Figure 1:**
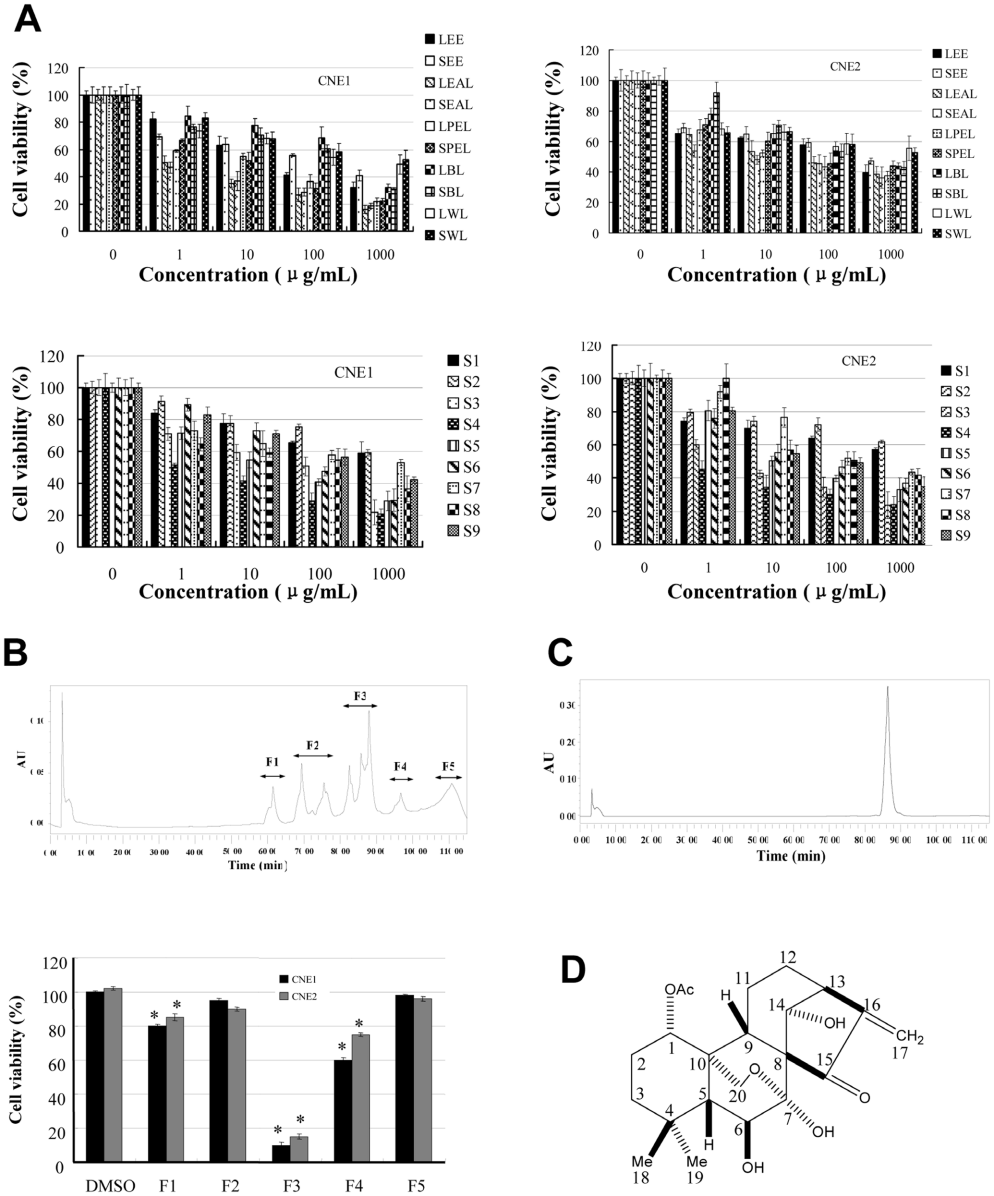
The anticancer activity-guided purification and identification of a C-20 oxygenated *ent*-kaurane from *R.serra*. (**A**), *R. serra* extracts and their subfractions inhibited NPC cell proliferation. Leaf ethanolic extract (LEE), stem ethanolic extract (SEE), leaf petroleum ether layer (LPEL), stem petroleum ether layer (SPEL), leaf ethyl acetate layer (LEAL), stem ethyl acetate layer (SEAL), leaf butanol layer (LBL), stem butanol layer (SBL), leaf water layer (LWL), stem water layer (SWL). After 72 hr treatment, cell viability was determined by the MTT assay. NPC cells treated with the vehicle control (DMSO) were used as the reference group with cell viability set at 100%. (**B**), S3 was analyzed by HPLC at 254 nm. F3 possessed the most effectively inhibitory effects on NPC cells. After 72 hr treatment, cell viability was determined by the MTT assay. NPC cells treated with the vehicle control (DMSO) were used as the reference group. (**C**), **Compound 1** was analyzed by HPLC at 254 nm. (**D**), The structure of **compound 1** was shown. The figures are representative of three experiments. The data are presented as mean ± S.D. of three separate experiments. * *P*<0.05, the significant differences between treatment and control groups.

The ethanolic extracts of *R. serra* leaf and stem were prepared by a refluxing method according to our previous study [24]. The ethanolic extracts were sequentially partitioned into the petroleum ether, ethyl acetate, butanol and water layers, respectively. Then the petroleum ether, ethyl acetate, butanol and water layers of the ethanolic extracts of *R. serra* leaf and stem were evaporated to dryness using a rotary evaporator (RE52AA, Yarong Equipment Co., Shanghai, China) under reduced pressure at 55 °C, respectively, to obtain eight subfractions [24]. The ethanolic extracts and their subfractions (including the petroleum ether, ethyl acetate, butanol and water layers) were completely dried in a vacuum oven overnight to remove the residual solvents, respectively. One hundred milligrams of each sample was weighed. And they were dissolved in 1 mL of DMSO, respectively. Then, the solutions were subjected to the MTT assays (Figure 1A). NPC cells treated with the vehicle control (DMSO) were used as the reference group. The final concentration of DMSO in the cell culture was 1%. The ethyl acetate layers of *R.serra* leaf and stem that showed the strongest inhibitory effects on proliferation of NPC cells were considered as the most active fractions. Then, the ethyl acetate layers were combined and chromatographed through a silica gel column (100×1000 mm). A gradient elution system of chloroform/methanol (1∶0–0∶1, v/v) was used to obtain nine fractions (S1–S9). The fractions (S1–S9) were completely dried in a vacuum oven overnight to remove the residual solvents, respectively. Then, the MTT assays were applied for the evaluation of the inhibitory effects of S1–S9 (dissolved in DMSO) on NPC cells (Figure 1A). S3, exhibiting the strongest inhibitory effects on CNE1 and CNE2 cells, was analyzed by HPLC (Figure 1B). Then it was separated by a sepacore chromatography system (Buchi labortechnik, Flawil, Switzerland) consisting of a pre-packed ODS-BP gel column (40×80 mm). A gradient system of water/methanol (9∶1–0∶1, v/v) was used to yield F1, F2, F3, F4, and F5 according to Figure 1B. Five fractions were completely dried in a vacuum oven overnight to remove the residual solvents, respectively. Then, F1, F2, F3, F4, and F5 (dissolved in DMSO) were applied for the MTT assays (Figure 1B). F3 possessing the most effectively inhibitory effects on NPC cells was further applied on a Sephadex LH-20 gel column (20×700 mm) to obtain **compound 1** (100 mg). **Compound 1** was completely dried in a vacuum oven overnight to remove the residual solvents. It was detected by HPLC (Figure 1C). Then, it was identified as lasiodin by 1D NMR spectra and electrospray ionization (ESI) mass spectrometry.

### Cellular morphology observation

NPC cells were treated with lasiodin at the indicated doses for 24 hr. NPC cells treated with the vehicle control (DMSO) were used as the reference group. The final concentration of DMSO in the cell culture was 1%. The cellular morphology was observed by an Olympus microscope fitted with a digital camera.

### Colony formation assay

About 600 cells were added to each well of the 6-well culture plates, and each group contained three wells. NPC cells were treated with the indicated doses of lasiodin. NPC cells treated with the vehicle control (DMSO) were used as the reference group. The final concentration of DMSO in the cell culture was 1%. After incubation at 37 °C for 14 days, the cells were washed twice with PBS and stained with crystal violet staining solution. The number of colony containing 50 cells was counted under a microscope. And the plate colony formation efficiency was calculated using the formula: plate colony formation efficiency  = (number of colony/number of cells inoculated) ×100%.

### Scratch assay

The scratch assay was performed to detect cell migration. The cells were grown to full confluence in the 6-well plates and incubated overnight in the starvation medium. The cell monolayers were wounded with a sterile pipette tip (200 µl) to make the gap or wounding space between NPC cells monolayers, and washed with PBS to remove the detached cells. The gaps were photographed using an Olympus microscope fitted with a digital camera. Then, the cells were treated with the indicated doses of lasiodin in the full medium and kept in a CO_2_ incubator. NPC cells treated with the vehicle control (DMSO) were used as the reference group. The final concentration of DMSO in the cell culture was 1%. After 24 hr, the medium was replaced with PBS. And the wound gaps were observed and photographed.

### Apoptosis assay

NPC cells (5×10^4^) were seeded in each well of the 6-well culture plate. NPC cells were treated with the indicated doses of lasiodin. NPC cells treated with the vehicle control (DMSO) were used as the reference group. The final concentration of DMSO in the cell culture was 1%. After 24 hr, the adherent and floating cells were collected and stained with Annexin V-FITC and propidium iodide (PI) using the Annexin V-FITC apoptosis detection kit (Invitrogen, Carlsbad, CA) per instruction of the manufacturer. Ten thousand cells per treatment were analyzed using a flow cytometer (BD Biosciences, Bedford, MA, USA). The apoptotic ratio was calculated in terms of the FITC-positive values of the cells including the early apoptotic cells (Annexin V-FITC-positive, PI-negative cells) and late apoptotic cells (Annexin V-FITC -positive, PI-positive cells).

### Western blot analysis

NPC cells were treated with lasiodin at the indicated doses for 24 hr. NPC cells treated with the vehicle control (DMSO) were used as the reference group. The final concentration of DMSO in the cell culture was 1%. And the cell lysates were subjected to Western blotting using the antibodies of GAPDH, p50, p52, p65, p68, p75 NF-κB, cytochrome-C, and Apaf-1 from Santa Cruz Biotechnology (Santa Cruz, USA). Moreover, the cell lysates were also subjected to Western blotting using the antibodies of caspase-3, cleaved caspase-3, caspase-9, cleaved caspase-9, intact PARP, cleaved PARP, Bcl-2, Bax, ERK 1/2, phospho-ERK 1/2, p38, phospho-p38, JNK, phospho-JNK, AKT, phospho-AKT, phospho-PI3K, and COX-2 from Cell Signaling Technology (Beverly, MA, USA).

### Confocal immunofluorescence

NPC cells grown on the chamber slides were incubated with lasiodin at 37°C for 12 hr and washed with PBS. And then, the cells were fixed for 30 min at room temperature with 4% paraformaldehyde. Subsequently, the cells were permeabilized in PBS with 0.2% Triton X-100. In order to study the localization of NF-κB, the cells were incubated with 1∶50 of rabbit anti-p65 antibody, and then incubated with 1∶100 of anti-rabbit IgG-fluorescein isothiocyanate (Zymed, San Francisco, CA). In order to study the localization of cytochrome-C, the cells were incubated with mouse anti-Cyto-C antibody (BD Biosciences, San Jose, CA, USA), and then incubated with rhodamine-conjugated goat anti-mouse IgG (Santa Cruz Biotechnology). In order to study the localization of Apaf-1, the cells were incubated with mouse anti-Apaf-1 antibody (Santa Cruz Biotechnology), followed by incubation with anti-rabbit IgG-fluorescein isothiocyanate (Zymed, San Francisco, CA). The cell nuclei were stained with 4′, 6-diamidino-2-phenylindole (DAPI), and the cells were examined under a fluorescence microscope (Carl Zeiss, Oberkochen, Germany). NPC cells treated with the vehicle control (DMSO) were used as the reference group. The final concentration of DMSO in the cell culture was 1%.

### DNA–protein binding by the streptavidin-agarose pulldown assay

The transactivator binding to a COX-2 core promoter probe was determined by a streptavidin-agarose pulldown assay as previously described [25]. A 479-bp biotin-labeled double-stranded probe corresponding to the COX-2 promoter sequence (−30 to −508) was used for the experiment. The DNA-bound NF-κB was analyzed by Western blotting. NPC cells treated with the vehicle control (DMSO) were used as the reference group. The final concentration of DMSO in the cell culture was 1%.

### Statistical analysis

The significance was evaluated by the paired *t* test. SPSS11.0 software (SPSS Inc., Chicago, USA) was used for all statistical analysis. All the experiments were done in triplicate, and the mean values and standard deviation were calculated.

## Results

### The purification and identification of an antiproliferative compound from R. Serra

To purify and identify the antiproliferative compounds from *R. serra*, we performed an activity-guided isolation and obtained a colorless crystal (MeOH) named **compound 1.** Its chemical structure was further identified by ESI-MS and NMR analysis. The molecular formula was established as C_22_H_30_O_7_ from its negative ESI-MS spectra (*m/z* 405.0 [M-H]^−^). The ^1^H and ^13^C NMR data are shown in Table 1. The NMR data suggested that **compound 1** exhibited the signals for three CH_3_, five CH_2_, six CH groups, four quaternary C-atoms, two olefinic C-atoms, one ketone C = O, and one ester C = O. In addition, ^1^H NMR spectra (*AB* system at δ 4.28 and δ 4.14) and ^13^C NMR spectra (hemiacetal group at δ 96.4) were in accordance with a 7, 20-epoxy-*ent*-kaur-16-en-15-one skeleton carrying one acetoxy group and three hydroxyl groups. Based on the above results and previous study [16], **compound 1** was identified as lasiodin. Its structure was shown in Figure 1D.

**Table 1:** ^1^H and ^13^C NMR data of lasiodin in CD_3_OD (δ in ppm).

Proton	δ_C_	δ_H_
1β	75.0	4.61, dd, 1H, *J* = 11.4, 5.2 Hz
2	24.2	
3	37.2	
4	32.6	
5	59.4	
6α	73.0	3.69, d, 1H, *J* = 6.4 Hz
7	96.4	
8	61.0	
9	52.2	
10	39.2	
11	17.3	
12	29.3	
13	42.8	2.99, d, 1H, *J* = 10.0 Hz
14β	72.4	4.88, d,1H, *J* = 1.2 Hz
15	207.8	
16	151.0	
17a	118.7	6.08, s, 1H
17b		5.53, s, 1H
18	31.1	1.11, s, 3H
19	20.2	1.06, s, 3H
20a	62.5	4.28, dd, 1H, *J* = 10.4, 1.2 Hz
20b		4.14, d, 1H, *J* = 10 Hz
OAc	169.8	
	19.4	1.93, s, 3H

### Lasiodin inhibited cell proliferation and migration

**Figure 2:**
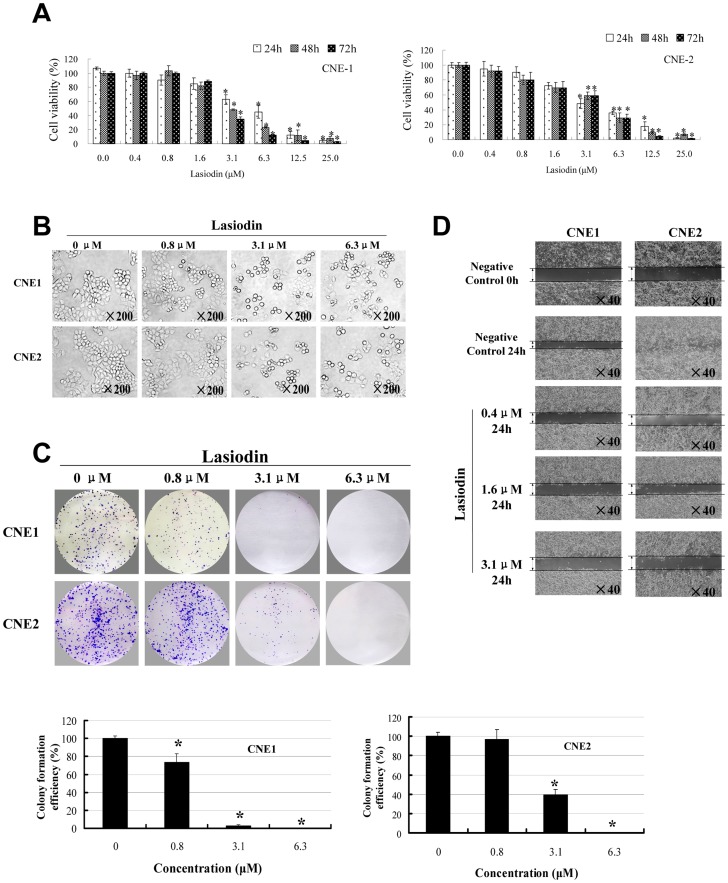
Lasiodin inhibited cell proliferation and migration. (**A**), Lasiodin inhibited NPC cell proliferation. The cells treated with the vehicle control (DMSO) were used as the reference group with cell viability set at 100%. (**B**), The cells were treated with lasiodin at the indicated does for 24 hr. The cellular morphology was observed by the phase contrast microscopy. NPC cells treated with the vehicle control (DMSO) were used as the reference group. (**C**), The colony formation efficiency was detected by the plate clone formation assay. After incubation for two weeks, the cells were stained with crystal violet staining solution. NPC cells treated with the vehicle control (DMSO) were used as the reference group. (**D**), Lasiodin mediated the NPC cell migration inhibition. Cell migration was analyzed by the scratch assay. CNE1 and CNE2 were grown to the full confluency. The cell monolayers were wounded with a sterile pipette tip, and washed with the medium to remove the detached cells from the plates. NPC cells were left either untreated or treated with the indicated doses of lasiodin. After 24 hr, the wound gap was observed and photographed. NPC cells treated with the vehicle control (DMSO) were used as the reference group. The figures are representative of three experiments. The data are presented as mean ± S.D. of three separate experiments. * *P*<0.05, the significant differences between treatment and control groups.

To further characterize the antiproliferative effects of lasiodin on NPC cells, the cells were cultured in the presence of the various concentrations of lasiodin (0.4–25 µM) for 24, 48 and 72 hr, respectively. And cell viability was analyzed by the MTT assays. As shown in Figure 2A, NPC cell viability was effectively inhibited by lasiodin in a dose-dependent manner. Additionally, the treatment of NPC cells with lasiodin also caused the distinct cellular morphological changes including rounding, shrinking, and detachment from the adjacent cells (Figure 2B). Moreover, lasiodin at 3.1 µM and 6.3 µM also significantly inhibited the colony formation efficiency of NPC cells (Figure 2C).

Next, we determined the effects of lasiodin on NPC cell migration. As shown in Figure 2D, the treatment with lasiodin effectively suppressed NPC cell migration. The gap or wounding space between the CNE2 cells layers was occupied completely by the migrating cells after 24 hr. However, the space between the lasiodin-treated cells was not occupied by the migrating cells. Nevertheless, no significant dose-related differences of the migration inhibition effects were found. These results were not consistent with the results of the cell viability and cell morphology assays. These results might be attributed to the different molecular mechanisms.

### Lasiodin induced apoptosis by activating the cytochrome-C/caspase signaling pathways

**Figure 3:**
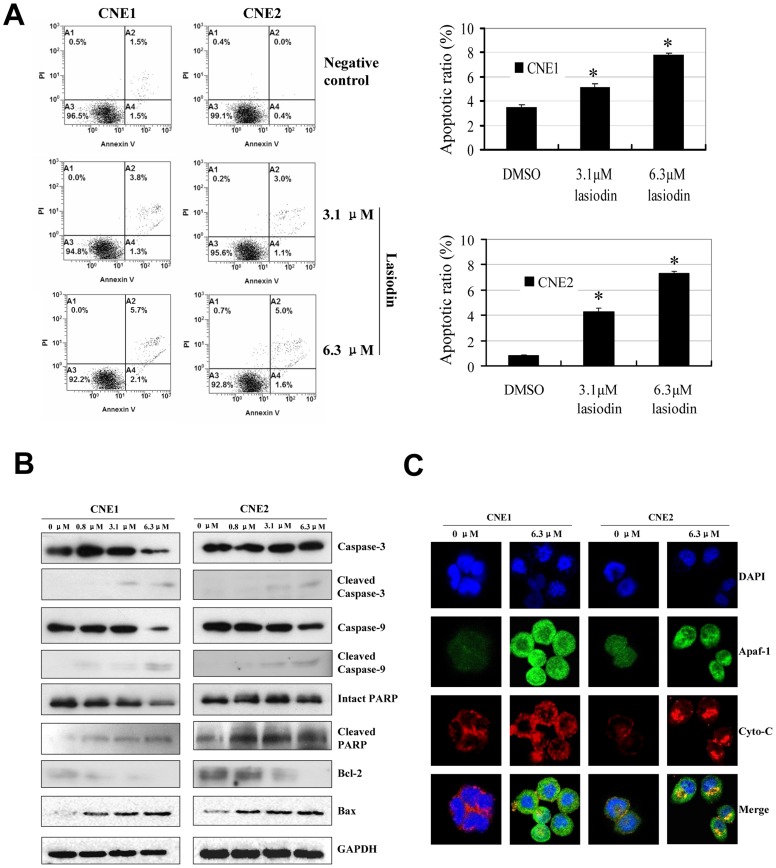
Activation of the caspase-dependent apoptotic pathway by lasiodin. (**A**), After treatment with lasiodin for 24 hr, both the adherent and floating cells were collected and stained with Annexin V-FITC and propidium iodide (PI). The apoptotic ratio was calculated in terms of the FITC-positive values of the cells. The apoptotic ratio was represented by the relative percentages of the FITC-positive cells versus the DMSO-treated cells (vehicle control). (**B**), CNE1 and CNE2 cells were treated with lasiodin at the doses of 0.8, 3.1, and 6.3 µM. After 24 hr treatment, the expressions of caspase-3, caspase-9, PARP, Bax, and Bcl-2 were determined, and the cleavage products of caspase-9, caspase-3, and PARP were detected by Western blotting. NPC cells treated with the vehicle control (DMSO) were used as the reference group. GAPDH was used as the control for sample loading. (**C**), CNE1 and CNE2 cells were treated with lasiodin at 6.3 µM. After 24 hr treatment, the releases of Cyto-C and Apaf-1 were determined by immunofluorescence imaging analysis to monitor the Cyto-C and Apaf-1 releases from inter-mitochondrial space into cytosol. NPC cells treated with the vehicle control (DMSO) were used as the reference group. The figures are representative of three experiments. The data are presented as mean ± S.D. of three separate experiments. * *P*<0.05, the significant differences between treatment and control groups.

The effect of lasiodin on apoptosis was analyzed. At the dose of 3.1 µM, lasiodin induced 5.1% and 4.3% apoptosis in CNE1 and CNE2 cells (Figure 3A), respectively, after 24 hr treatment. However, the use of lasiodin at 6.3 µM for 24 hr resulted in the induction of 7.8% and 7.3% apoptosis in CNE1 and CNE2 cells (Figure 3A), respectively.

To determine the mechanisms of lasiodin-induced apoptosis, we detected the effects of lasiodin on the expressions of three key apoptosis-related proteins including caspase-3, caspase-9, protease poly ADP-ribose polymerase (PARP) and their cleaved proteins in CNE1 and CNE2 cells after 24 hr treatment. As seen in Figure 3B, the treatment with lasiodin at the doses of 3.1 µM and 6.3 µM resulted in the induction of the cleaved caspase-3, caspase-9 and PARP proteins. These results suggested that lasiodin could modulate the activation of the caspase apoptotic signaling pathways in NPC cells.

The anti-apoptotic Bcl-2 protein is a potent antagonist of the mitochondrial pathway of apoptosis. Bax exerts the pro-apoptotic activity by the translocation from cytosol to mitochondria. And it induces the cytochrome-C (Cyto-C) release. We detected the Bcl-2 and Bax expressions in lasiodin-treated NPC cells. As shown in Figure 3B, the treatment with lasiodin dramatically downregulated the Bcl-2 protein expression in a concentration-dependant manner in NPC cells. However, the treatment with lasiodin at 3.1 µM and 6.3 µM considerably promoted the expression of Bax. These results indicated that lasiodin also modulated the expressions of the Bcl-2 family proteins to facilitate apoptosis.

Cyto-C is an upstream molecule of the Apaf-1-mediated caspase-dependent apoptosis pathways. And it leads to the caspase-9 activation and apoptosis [26]. Many apoptotic stimuli induce the Cyto-C release from mitochondrial inter-membrane space into cytosol, thereby inducing apoptosis [27]. Therefore, we performed immunofluorescence imaging (IFI) analysis to monitor the changes in the subcellular localization of Cyto-C in lasiodin-treated cells. As shown in Figure 3C, lasiodin effectively promoted the release of Cyto-C from inter-mitochondrial space into cytosol in CNE1 and CNE2 cells.

Apaf-1 can form a complex with caspase-9 in the presence of Cyto-C. The formation of the complex results in the activation of caspase-9 and caspase-3, and subsequently induces apoptosis [28]. We also analyzed the effect of lasiodin on the expression of the Apaf-1 protein in NPC cells by IFI analysis. The treatment with lasiodin triggered the expression of Apaf-1 in NPC cells. These data suggested that lasiodin could trigger the release of Cyto-C from inter-mitochondrial membrane space into cytosol and facilitate the downstream Apaf-1-mediated caspase-dependent apoptosome assembly and caspase activation in NPC cells.

### Lasiodin reduced the activity of the AKT signaling pathways

**Figure 4:**
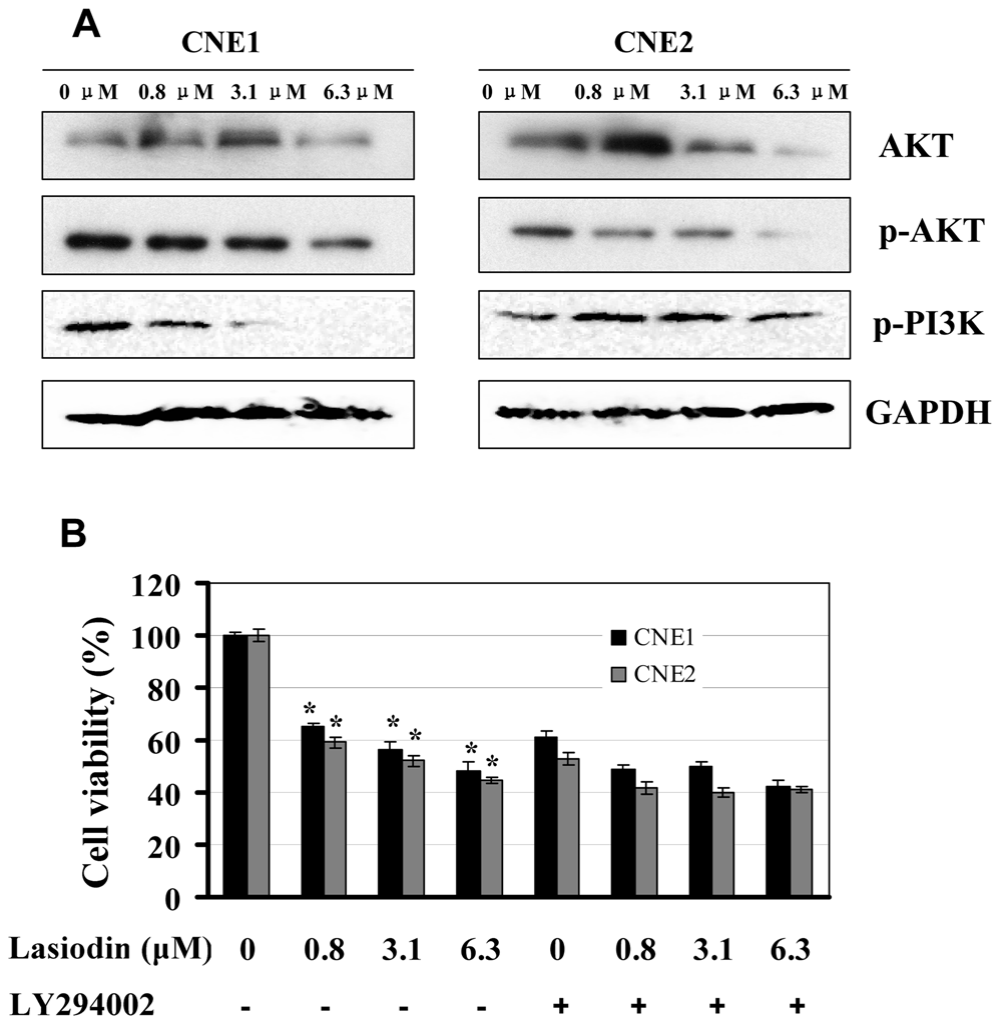
Inhibition of PI3K/AKT signaling by lasiodin. (**A**), CNE1 and CNE2 cells were treated with lasiodin at the indicated doses. After 24 hr treatment, the expressions of the phosphorylated or total protein of AKT and PI3K were detected by Western blotting. NPC cells treated with the vehicle control (DMSO) were used as the reference group. GAPDH was used as the control for sample loading. (**B**), CNE1 and CNE2 cells were treated with the AKT-selective inhibitor (LY294002, 5 µM) for 4 hr, and then treated with lasiodin at the indicated doses. After 48 hr treatment, cell viability was determined by the MTT assay. The figures are representative of three experiments. The data are presented as mean ± S.D. of three separate experiments. * *P*<0.05, the significant differences between treatment groups without the inhibitor and control groups without the inhibitor. # *P*<0.05, the significant differences between treatment groups with the inhibitor and control groups with the inhibitor.

We determined whether lasiodin was capable of regulating the inactivation of the PI3K/AKT signaling pathways in NPC cells. The significant reduction of the AKT, phosphorylated PI3K and AKT proteins was observed in a dose-dependent manner (Figure 4A), respectively. In addition, we analyzed the effects of the PI3K/AKT-selective inhibitor (LY294002) on the inhibition of lasiodin-mediated proliferation to further confirm the inactivation of the PI3K/AKT signaling pathways. As shown in Figure 4B, the treatment with LY294002 in CNE1 and CNE2 cells suppressed cell proliferation. And cell viability of NPC cells that pretreated with LY294002 and then treated with lasiodin was slightly decreased. Thus, the treatments of lasiodin at different concentrations did not lead to the significant enhancement of the proliferation inhibition of the cells pretreated with the inhibitor. These results confirmed the important role of lasiodin in modulating the PI3K/AKT signaling pathways.

### Lasiodin affected the activity of the MAPK signaling pathways

**Figure 5:**
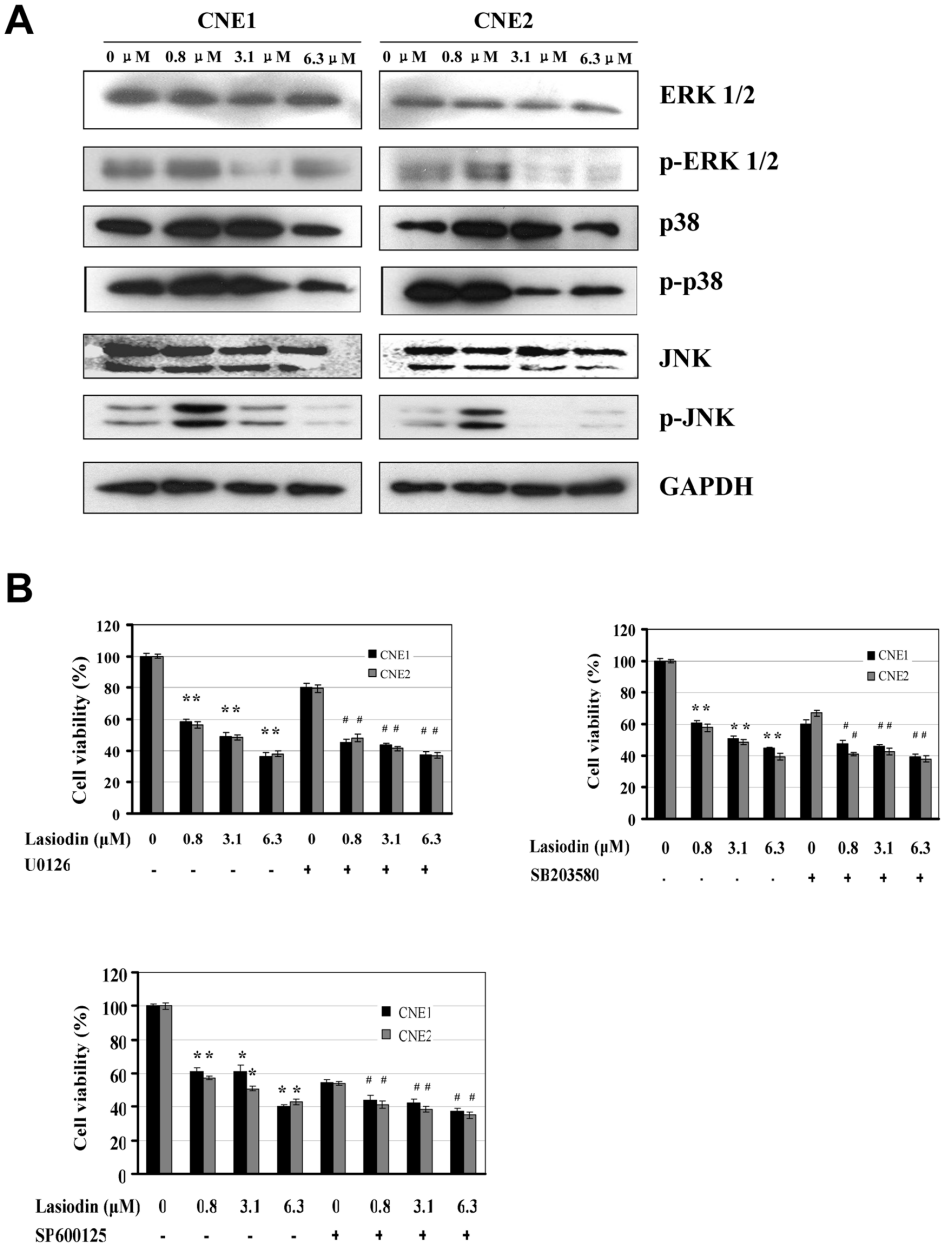
Supression of MAPK signaling by lasiodin. (**A**), CNE1 and CNE2 cells were treated with lasiodin at the indicated doses. After 24 hr treatment, the expressions of the phosphorylated or total protein of ERK1/2, JNK and p38 were detected by Western blotting. NPC cells treated with the vehicle control (DMSO) were used as the reference group. GAPDH was used as the control for sample loading. (**B**), CNE1 and CNE2 cells were treated with the ERK-selective inhibitor (U0126, 20 µM), JNK inhibitor (SB203580, 600 nM) and p38 inhibitor (SP600125, 2.5 µM) for 4 hr, respectively, and then treated with lasiodin at the indicated doses. After 48 hr treatment, cell viability was determined by the MTT assay. The figures are representative of three experiments. The data are presented as mean ± S.D. of three separate experiments. * *P*<0.05, the significant differences between treatment groups without the inhibitor and control groups without the inhibitor. # *P*<0.05, the significant differences between treatment groups with the inhibitor and control groups with the inhibitor.

As shown in Figure 5A, lasiodin had insignificant effect on the expression of ERK 1/2. Lasiodin (0.8 µM) upregulated the expressions of the p38 and p-JNK proteins. However, the treatments with lasiodin at the doses of 3.1 µM and 6.3 µM significantly reduced the expressions of the phosphorylated ERK 1/2 and JNK proteins in CNE1 and CNE2 cells. Lasiodin also significantly downregulated the expression of p-p38 in CNE2 cells. Nevertheless, lasiodin only slightly decreased the expression of p-p38 in CNE1 cells. These results implied that the lasiodin-mediated proliferation inhibition was attributed to the inactivation of the MAPK signaling pathways.

To confirm the involvement of the MAPK signaling pathways in the lasiodin-mediated proliferation inhibion of NPC cells, we analyzed the effects of the ERK-selective inhibitor (U0126, 20 µM), JNK inhibitor (SB203580, 600 nM) and p38 inhibitor (SP600125, 2.5 µM) on the lasiodin-mediated proliferation inhibition by the MTT assays. As shown in Figure 5B, the proliferation inhibtion activity was moderately affected by U0126 at 20 µM. The combination of U0126 and lasiodin significantly decreased cell viability. The results implied that the activation of the ERK1/2 signaling pathways was partially involved in lasiodin-induced apoptosis. However, the JNK inhibitor SB203580 and p38 inhibitor SP600125 dramatically decreased cell viability in CNE1 and CNE2 cells (Figure 5B). In addition, the pretreatments with the JNK inhibitor and p38 inhibitor slightly affected the lasiodin-mediated proliferation inhibition in NPC cells. These results indicated that the JNK and p38 signaling pathways played important roles in mediating the lasiodin-induced proliferation suppression.

### Lasiodin suppressed the COX-2 expression and inhibited NF-κB binding

**Figure 6:**
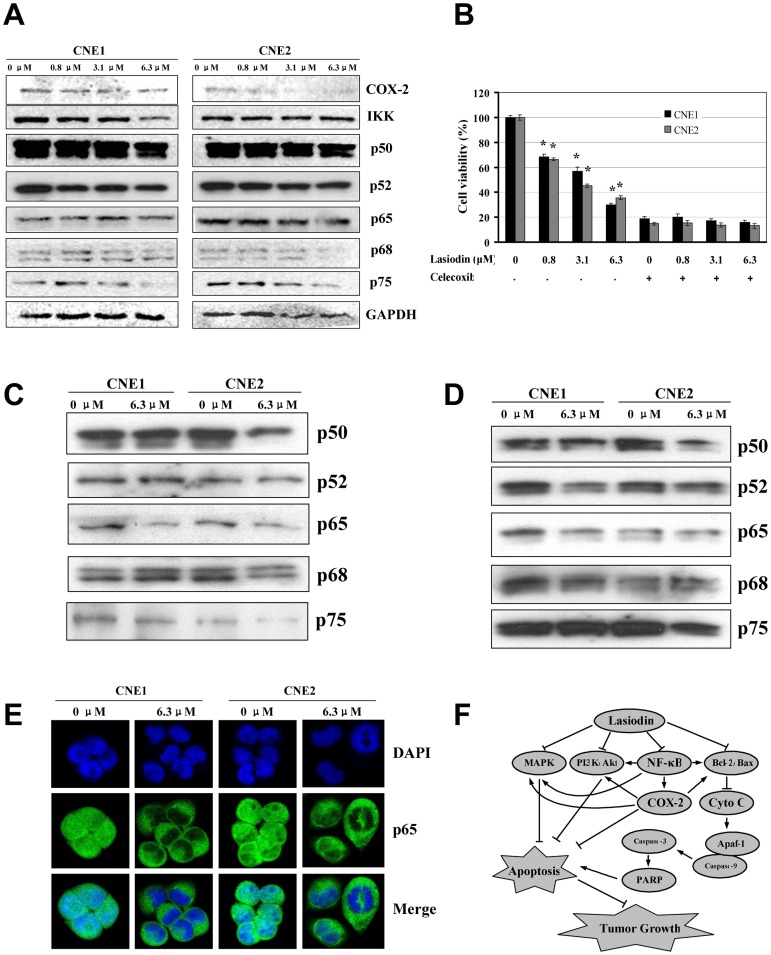
Suppression of COX-2 expression and NF-κB binding by lasiodin. (**A**), CNE1 and CNE2 cells were treated with lasiodin at the indicated doses. After 24 hr treatment, the expressions of COX-2, IKK and NF-κB were detected by Western blotting. NPC cells treated with the vehicle control (DMSO) were used as the reference group. GAPDH was used as the control for sample loading. (**B**), CNE1 and CNE2 cells were treated with the COX-2-selective inhibitor (celecoxib, 20 µM) for 4 hr, and then treated with lasiodin at the indicated doses. After 48 hr treatment, cell viability was determined by the MTT assay. (**C**), CNE1 and CNE2 cells were treated with lasiodin at 6.3 µM for 24 hr. The binding of the transactivators to the COX-2 promoter was analyzed by the streptavidin-agrose pulldown assay. NPC cells treated with the vehicle control (DMSO) were used as the reference group. (**D**), CNE1 and CNE2 cells were treated with lasiodin at the dose of 6.3 µM for 24 hr. The nuclear extracts were prepared, and NF-κB was detected by Western blotting. NPC cells treated with the vehicle control (DMSO) were used as the reference group. (**E**), CNE1 and CNE2 cells were treated with lasiodin at 6.3 µM for 24 hr. The NF-κB nuclear translocations in CNE1 and CNE2 cells were determined by immunofluorescence imaging analysis. NPC cells treated with the vehicle control (DMSO) were used as the reference group. (**F**), The proposed mechanisms were shown. The figures are representative of three experiments. The data are presented as mean ± S.D. of three separate experiments. * *P*<0.05, the significant differences between treatment groups without the inhibitor and control groups without the inhibitor. # *P*<0.05, the significant differences between treatment groups with the inhibitor and control groups with the inhibitor.

COX-2 and NF-κB play important roles in tumorigenesis. We detected the effect of lasiodin on the COX-2 expression in NPC cells. As shown in Figure 6A, the treatment with lasiodin suppressed the COX-2 protein expression in a concentration-dependent manner. In addition, we validated the role of lasiodin in regulating the COX-2 signaling pathways in CNE1 and CNE2 cells. As shown in Figure 6B, cell proliferation was significantly inhibited by the treatment with a COX-2 -selective inhibitor (celecoxib, 20 µM). However, lasiodin did not dramatically alter the inhibition mediated by celecoxib. These results implied that the COX-2 inhibitor was efficient to inhibit proliferation of NPC cells. And the inhibition of NPC cells proliferation by lasiodin was mediated by the inactivation of the COX-2 signaling pathways.

NF-κB could protect the cells from apoptosis through up-regulating the p38-NF-κB survival pathways [29], [30]. To evaluate the effects of lasiodin on the regulation of the NF-κB expression in CNE1 and CNE2 cells, we detected the effects of lasiodin on the expressions of p50, p52, p65, p68 and p75 NF-κB. As seen in Figure 6A, the treatment with lasiodin had insignificant effects on the expressions of p50, p52 and p65. However, the treatment with lasiodin at 3.1 µM and 6.3 µM downregulated the expressions of p68 and p75. Furthermore, IκB kinase complex (IKK) decreased in CNE1 cells treated with lasiodin. However, there were insignificant changes of IKK in CNE2 cells treated with lasiodin.

The binding activities of the transactivators on the COX-2 gene promoter modulate the transcription activation of COX-2. Then, we tested the effects of lasiodin on the binding activity of NF-κB to the COX-2 promoter in NPC cells. A biotin-labeled 479-bp COX-2 promoter region corresponding to the 5-flanking sequence of the human COX-2 gene from −30 to −508 was used as the probe to assess the binding of the transactivators to the COX-2 promoter by the streptavidin-agarose pulldown assay. Nuclear extracts of NPC cells treated with lasiodin were incubated with the biotin-labeled COX-2 promoter probes and streptavidin-conjugated agarose beads. The biotin-streptavidin complexes were pulled down. The p50, p52, p65, p68 and p75 proteins in the pulldown complexes were analyzed by Western blotting. The results showed that lasiodin significantly inhibited the binding of p50, p65 and p75 to the COX-2 promoter probe (Figure 6C). The treatment with lasiodin also slightly downregulated the expressions of p50, p52, p68 and p75 in nuclear (Figure 6D). However, the expression levels of p65 in nuclei of lasiodin-treated NPC cells (especially CNE1) were obviously lower than those of p65 in nuclei of DMSO-treated cells. Moreover, we performed IFI analysis to confirm the nuclear localization of p65 in CNE1 and CNE2 cells. The treatment with lasiodin caused the translocation of p65 from nuclei to cytoplasm (Figure 6E). The results suggested that the inhibition of NPC cell proliferation by lasiodin might be mediated by potentiating the NF-κB translocation form nuclei to cytoplasm. This is consistent with our previous observation (Figure 6D). These results also suggested that the NF-κB signaling pathways might be involved in the lasiodin-mediated suppression of COX-2 and cell proliferation in NPC cells.

## Discussion

Phytochemicals from the dietary and medicinal plants have emerged as the promising sources of the potential anticancer agents [31]. *R. serra* possessed several diterpenoids including C-20 oxygenated *ent*-kauranes, 6, 7-seco-*ent*-kauranes, abietanes and *ent*-abietanes [13], [14], [32]. And these diterpenoids exhibited strong anti-proliferation effects on human cancer cells [16], [33]-[35]. In the current study, lasiodin was obtained through an antiproliferative activity-guided assay. Furthermore, we found that lasiodin caused the distinct cellular morphological changes. It also significantly suppressed colony formation and effectively suppressed cell migration. The strong antiproliferative activity of lasiodin is related to its chemical structure. The chemical structure refers to a α-methylenecyclopentanone moiety, a hydroxy group at position 6, a carbonyl group at position 15 [35], [36], a β-OH group at position 14 and a hydroxy group at position 7 [16].

We also found that lasiodin regulated several signaling pathways including the cytochrome-C/caspase-dependent-apoptotic signaling pathways, the PI3K/AKT and MAPK signaling pathways, and the COX-2 signaling pathways (Figure 6F). To the best of our knowledge, it is the first time that the treatment with lasiodin on NPC cells and the underlying mechanisms have been reported. The results could serve as a basis for guiding the treatment with the natural antiproliferative compounds for improving the efficiency of NPC therapy.

The COX-2 expression has been shown to upregulate the PI3K and ERK signaling pathways. And the overexpression of COX-2 can induce angiogenesis, cell proliferation and invasion [27]. In the present study, we observed that the treatment with lasiodin mediated the suppressions of the COX-2, PI3K and MAPK signaling pathways in NPC cells and resulted in the cell growth suppression and apoptosis induction. These results implied that the inhibition of COX-2 by lasiodin could downregulate the PI3K and MAPK signaling pathways in NPC cells.

The transcription factor NF-κB includes five members including NF-κB1 (p50), NF-κB2 (p52), RelA (p65), RelB (P68) and c-Rel (p75). In addition, NF-κB controls the critical genes including COX-2, Bcl-2, and genes required for invasion and angiogenesis such as MMP9 and VEGF in the early and late stages of aggressive cancer [37], [38]. NF-κB can be activated by a variety of stimuli [39], [40]. The stimulation of the cells results in the phosphorylation of the NF-κB/IκB complex by IKK and the subsequent degradation of the IκB protein. NF-κB enters nuclei upon the degradation of IκB. In the study, the downregulation of IKK in lasiodin-treated CNE1 cells was observed. However, lasiodin did not significantly inhibit IKK in CNE2 cells.

To date, a large number of the natural compounds have been reported as NF-κB inhibitors [41], [42]. Four diterpenoids including two 7,20-epoxy-ent-kaurenoids (oridonin and ponicidin), and two C-20-nonoxygenated-ent-kauranoids (xindongnin A and xindongnin B) were found to be the potent inhibitors of NF-κB [40]. Our study also indicated that the diterpenoids were a new class of the NF-κB inhibitors. It is well-known that p65 is a critical transactivation subunit of NF-κB [43], [44]. However, those diterpenoids including oridonin, ponicidin, xindongnin A and xindongnin B did not significantly block the nuclear translocation of p65 [40]. However, we observed the complete accumulation of p65 in cytoplasm in lasiodin-treated NPC cells, especially in CNE1 cells.

In summary, we demonstrated that lasiodin exhibited the antiproliferative, proapoptotic, anti-invasive, and antiangiogenic effects through simultaneous modulation of the cytochrome-C/caspase, AKT/MAPK, and COX-2/NF-κB signaling pathways. Our results provided the new insights into the molecular mechanisms of the lasiodin-mediated NPC cell suppression. The results also suggested that lasiodin was a natural agent for the prevention and treatment of NPC.
